# Alcohol related cognitive impairments in patients with and without cirrhosis

**DOI:** 10.1192/j.eurpsy.2023.351

**Published:** 2023-07-19

**Authors:** B. Angerville, M.-A. Jurdana, R. Sarba, É. Nguyen-Khac, M. Naassila, A. Dervaux

**Affiliations:** ^1^Filière universitaire d’addictologie, EPS Barthélemy Durand, Etampes; ^2^GRAP U 1247, INSERM-Université Picardie Jules verne; ^3^ Université Picardie Jules Verne; ^4^Département d’Hépato-Gastroenterologie, CHU d’Amiens, Amiens; ^5^UR PSYCOMADD, Université Paris saclay-Hopital Paul Brousse, Villejuif, France

## Abstract

**Introduction:**

Up to 80 % of patients with alcohol use disorders (AUD) display cognitive impairments. Some studies suggested that cognitive functions could be worsened by hepatic damage, particularly cirrhosis. Cirrhosis is widespread in patients with AUD, indeed one third of them develop cirrhosis during their lifetime (Zhang et al. Alcohol Clin Exp Res. 2022). Currently, patients treated for cirrhosis do not benefit from a systematic assessment of alcohol related cognitive impairments. The Brief Screening Tool for Alcohol-Related Neuropsychological Impairments (BEARNI) is a specific tool developed to screening for those impairments.

**Objectives:**

The primary objective of this study was to compare BEARNI mean scores in a group of AUD patients with (AUD/C+) or without cirrhosis (AUD/C-).

**Methods:**

We conducted a prospective, monocentric study at the Amiens University Hospital. Subjects were consecutively recruited from the hepato-gastroenterology department of Amiens University hospital and from the local substance abuse treatment department. All patients were assessed using BEARNI test, demographical (age, gender, number of years of scholarship), and clinical variables, using Child-Pugh scores and Alcohol use disorders identification test (AUDIT). The BEARNI mean score in the AUD/C+ group was compared to the mean score in the AUD/C- group using an Analysis of covariance (ANCOVA) with age and educational level as covariate. Between group comparisons were performed using post hoc analysis with Tukey HSD test.

**Results:**

107 patients (75 AUD/C+, 32 AUD/C-) were included in this study. AUD/C- patients were significantly younger than AUD/C+ patients (respectively, 45.5 ± 6.8 vs 59.3 ± 9.3; p<0.0001). There were no differences regarding gender and years of scholarship. Child-Pugh mean scores were 6.9 ± 2.4 in the AUD/C+ group. AUDIT mean scores were significantly lower in the group of patients with AUD and cirrhosis than in the group of patients with AUD without cirrhosis. After adjusting on age and educational level, we found that mean BEARNI total and cognitive scores in the group of patients with AUD and cirrhosis were significantly lower than in the group of patients with AUD without cirrhosis (respectively, 13.8 ± 0.7 vs 7.8± 0.4 F=46.8; p<0.0001 and 10.6 ± 0.6 vs 6.9± 0.3; F=30.1; p<0.0001). The mean subscores of delayed verbal memory, alphabetical ordination, alternating verbal fluency and ataxia subtests were also significantly lower in the group of group of patients AUD/C + (respectively, 1.8 ± 0.1 vs 2.8 ± 0.2, F= 13.9, p<0.0001; 1.8 ± 0.1 vs 2.6 ± 0.2, F= 10.6, p<0.0001; 2.4 ± 0.1 vs 3.6 ± 0.2, F= 13.4, p<0.0001; 0.9 ± 0.2 vs 3.1 ± 0.2, F= 30.6, p<0.0001).

**Conclusions:**

In the present study, the patients with AUD and cirrhosis had more cognitive impairments than their counterparts without cirrhosis. Longitudinal studies are needed to investigate how cirrhosis can influence cognitive impairments.

**Disclosure of Interest:**

None Declared

